# Indirect costs constitute a major part of the total economic burden of obesity: a Finnish population-based cohort study

**DOI:** 10.1186/s12889-025-22978-9

**Published:** 2025-05-10

**Authors:** Aino Vesikansa, Juha Mehtälä, Susanna Aspholm, Kirsi Kallio-Grönroos, Katja Mutanen, Annamari Lundqvist, Tiina Laatikainen, Tero Saukkonen, Kirsi H. Pietiläinen

**Affiliations:** 1 MedEngine Oy, Eteläranta 14, 00100 Helsinki, Finland; 2 Novo Nordisk Farma Oy, Espoo, Finland; 3 Finnish Institute for Health and Welfare, Helsinki, Finland; 4 Wellbeing Services County of North Karelia (Siun sote), Joensuu, Finland; 5 Institute of Public Health and Clinical Nutrition, University of Eastern Finland, Kuopio, Finland; 6 Obesity Research Unit, University of Helsinki, Helsinki, Finland

**Keywords:** Body-mass index, Obesity, Economic burden, Indirect costs, Direct costs, Total costs

## Abstract

**Background:**

The growing prevalence of overweight and obesity (OB) poses a considerable economic burden worldwide. However, nationally representative, detailed analyses estimating the total burden of OB are few. We characterized direct, indirect, and total costs of overweight and obesity in a population-based cohort of Finnish adult individuals and evaluated the additional total costs attributed to overweight and obesity.

**Methods:**

The study cohort included 5,587 randomly-selected individuals (≥18 years of age) who participated in the national FinHealth 2017 health examination survey. The main study group consisted of working-age individuals (18–64 years of age; *n* = 3,914). Individual-level data were collected from the nationwide registers by the Finnish Institute for Health and Welfare (healthcare resource utilization), Social Insurance Institution of Finland (prescription medications, sick leaves, disability pensions, rehabilitation periods), and Statistics Finland (deaths). Indirect costs were calculated using the Human Capital Approach, and direct costs were based on the medication purchases and healthcare resource use.

**Results:**

The mean annual indirect costs were €1,683 (SD, €6,395) per person for the working-age individuals with normal-weight (NW), €2,957 (€8,797) for individuals with overweight (OW), €4,488 (€11,607) for individuals with class I obesity (OBI), and €4,654 (€11,383) for individuals with class II–III obesity (OBII–III). The mean annual total (direct + indirect) costs were €3,314 (SD, €8,358) per person in the NW, €4,902 (€10,747) in the OW, €7,129 (€14,313) in the OBI, and €7,372 (€14,423) in the OBII–III groups. Compared with individuals with NW, OW was associated with 31% (rate ratio, RR, 1.31; 95% CI, 1.09–1.58; *p* = 0.005), OBI with 83% (RR, 1.83; 95% CI, 1.46–2.28; *p* < 0.001), and OBII–III with 95% (RR, 1.95; 95% CI, 1.48–2.55; *p* < 0.001) higher total costs in working-age individuals. When adjusted for age and sex, the predicted total annual cost difference per person was €1,124 for OW, €3,002 for OBI, and €3,443 for OBII–III compared with a person with NW.

**Conclusions:**

Indirect costs constitute a major part of the total costs of obesity in the working-age population. Compared with NW, the total costs are significantly higher not only for severe obesity, but also for OW and OBI.

**Supplementary Information:**

The online version contains supplementary material available at 10.1186/s12889-025-22978-9.

## Introduction

The worldwide prevalence of obesity has nearly tripled during the last 50 years, and approximately 2.6 billion people had overweight (body mass index, BMI, 25.0–29.9 kg/m^2^) or obesity (BMI, ≥ 30.0 kg/m^2^) in 2020 [1, 2]. The number is expected to rise above 4 billion by 2035, about half of the world’s population [1]. Today, obesity is acknowledged as a chronic, complex disease with underlying genetic, physiological, psychological, and environmental factors [3, 4]. Obesity is a risk factor for several comorbidities and decreased Quality of Life (QoL), and has been reported to reduce the life expectancy by 2.7 years across the population of the Organization for Economic Co-operation and Development (OECD) countries [2, 5–7].

The steady increase in obesity places a considerable burden on individuals, society, and the economy. Overweight and obesity had an estimated costs of US$1.96 trillion to the global economy in 2020 and reduced the global gross domestic product (GDP) by 2.4% [1, 2]. In the OECD countries, the treatment of obesity and related comorbidities is estimated to cost on average 8.4% of the total healthcare expenditure [2, 8]. In Finland, obesity was shown to be associated with 36% higher direct costs due to increased use of healthcare resources (HCRU) and prescribed medications, resulting in a predicted total direct annual cost difference of 626€ for an individual with obesity compared with an individual without [9].

Besides direct costs, obesity has a remarkable effect on the broader economy as it reduces labor force productivity and human capital [8]. Absenteeism, presenteeism, and premature mortality are all associated with BMI increase [10–13]. Several studies suggest that indirect costs arising from sick leaves, disability, reduced productivity, and premature mortality exceed the direct costs of obesity [13, 14]. Moreover, individuals with obesity have significantly lower gross earnings and higher social benefit transfers than individuals with normal weight [15, 16].

Despite the awareness of global obesity epidemic, only a few population-based studies have been conducted to estimate the total economic burden of obesity [12, 14–17]. Many available reports cover either direct or indirect costs, and varying methodologies utilizing literature search, registry, and self-reported data complicate the interpretation and generalizability of the findings [9–11, 13, 18–20]. Thus, up-to-date cost analyses are needed to understand the current burden of obesity, as well as to guide the development of health policies and allocate healthcare resources optimally [8].

In Finland, the nationally representative FinHealth health examination survey combined with comprehensive, nationwide health register data provide unique tools to assess the burden of obesity at the population-level [6, 9]. Finland had one of the largest overweight rate increases in the EU between 2014 and 2019, however, recent analyses on indirect and total costs of obesity are lacking [21]. The aim of this study was to characterize the association between BMI group and indirect costs related to sick leaves, work disability, and premature mortality in a population-based cohort of Finnish working-age individuals. Additionally, total (direct + indirect) costs of obesity in the Finnish working-age and total adult population were assessed at the individual and national level.

## Methods

### Study cohort and data collection

The study cohort was based on the national, cross-sectional FinHealth 2017 health examination survey conducted by the Finnish Institute for Health and Welfare (THL) in 2017, with the goal of producing information on health, health behavior, functional capacity, and well-being of adults in Finland [22]. For the FinHealth 2017 study, a representative sample of 10,305 individuals (≥ 18 years of age) were randomly selected from mainland Finland using a stratified one- and two-stage design [22, 23]. The data in the FinHealth study were gathered using health examination measurements and self-administered questionnaires. In the health examination, height and weight were measured using standardized methods, and blood and urine samples were collected. Of the recruited individuals, 59% participated in the health examination.

The FinHealth 2017 data were supplemented with data from nationwide health and social care registries (Supplementary Table 1). For determination of indirect costs, dates and causes of sick leaves (> 10 days), disability pensions, and rehabilitation periods were collected from the Social Benefits Register by The Social Insurance Institution of Finland (Kela) and data on the dates and causes of death were collected from the Cause of Death Register by Statistics Finland. For direct costs, data on primary and secondary public healthcare contacts and hospital stays with diagnoses (International Classification of Diseases – 10 th revision, ICD-10) were collected from the Care Register for Health Care and the Register of Primary Health Care Visits administered by the Finnish Institute for Health and Welfare. Data on purchases and costs of prescription medicines were collected from the Prescription Register by The Social Insurance Institution of Finland.

All register data were collected from the period covering one year prior and four years after (from 1st of April 2016 until 31st March 2021) the collection of the cross-sectional FinHealth 2017 data. Data linkage was performed using personal identification numbers.

### Study groups

The main analyses were restricted to the FinHealth 2017 study participants who had BMI measured in the health examination, were not underweight (BMI < 18.5 kg/m^2^, *n* = 34) and were of working age (18–64 years of age, *n* = 3,914) during the study period (Supplementary Fig. 1). In addition, supplementary analyses were conducted in the total adult population (≥ 18 years of age, *n* = 5,587). The following BMI subgroups were defined according to WHO’s classifications: 18.5–24.9 kg/m^2^ (normal weight, NW), 25.0–29.9 kg/m^2^ (overweight, OW), 30.0–34.9 kg/m^2^ (class I obesity, OBI), and ≥ 35.0 kg/m^2^ (class II–III obesity, OBII–III). In the selected analyses, results were further stratified by sex.

### Clinical characteristics

Presence of metabolic comorbidities (MetCs) was defined based on anthropometric, physiological, and laboratory measurements in the FinHealth 2017 health examinations (National Cholesterol Education Program’s Adult Treatment Panel III report [ATP III] criteria for metabolic syndrome components) [24], and on the purchases of selected medications data from the Prescription Register by the Social Insurance Institution of Finland as described in [25]. Presence of selected disease groups was defined based on ICD-10 codes recorded in primary and secondary healthcare (Supplementary Table 2).

### Outcomes

The primary outcome measures were annual indirect costs due to sick leaves (> 10 days; shorter sick leaves are paid by the employer and not available in national registers), disability pensions, rehabilitation periods and premature mortality (death occurring before the age of 65), and total costs including indirect and direct costs (primary and secondary public HCRU and purchases of prescription medications).

Secondary outcomes included individual components of indirect costs (percentage of individuals with sick leaves, disability pensions, rehabilitation periods, and premature mortality, length of a period, and associated annual costs), the most common causes for the indirect cost components (ICD-10 codes), and annual total costs extrapolated to the national level.

### Statistical analyses

In all analyses, the weighting of observations was used to match the distribution of known background factors (age, sex, etc.) in the group of study participants with that in the whole Finnish population. The weighting method used was the same as in the original FinHealth study [22]. The individual-level results were extrapolated to the national level using the extrapolative weights, which aim to have weighted number of persons that represents the whole Finland.

For the whole study population, follow-up period finished at death or at the end of study period, whichever came first, and indirect costs were considered zero from 65 years onwards. For the analyses of costs of premature mortality, the follow-up period finished the year patients would have turned 65 or the end of the study period, but not death. All outcome analyses were annualized over the whole follow-up period (i.e., for each person, the costs over the follow-up were divided by the follow-up time in years). A sensitivity analysis excluding the last year of follow-up (from 1st of April 2016 until 31st March 2020) was performed to estimate the potential impact of COVID-19 pandemic on costs. Categorical variables were summarized with numbers and proportions. Continuous variables were summarized with mean and standard deviation (SD). Costs of sick leaves, disability pensions, rehabilitation periods, and premature mortality were calculated using the Human Capital Approach (HCA), i.e., considering the age and sex-specific mean gross wages in Finland in 2019 and the days lost (based on the length of a period for paid benefits) due to morbidity and mortality [26, 27]. For sick leaves, a period of 10 working days was added to cover the non-deductible waiting period before the allowance is paid. The healthcare costs were calculated based on standard unit costs reported by THL [28].

A zero-inflated, age and sex-adjusted overdispersed Poisson approach was used to evaluate the association between the BMI group and indirect costs. The approach consisted of two models: i) a logistic regression model for the risk of having non-zero indirect costs, and ii) a Poisson regression model for positive costs. The associations between the BMI group and direct and total costs were estimated using an age- and sex-adjusted overdispersed Poisson regression model. Regression modeling results were reported by estimated coefficients, 95% confidence intervals (CI), and *p*-values.

## Results

### Demographic and clinical characteristics

Based on the FinHealth 2017 study, 35.8% of the working-age Finnish population had BMI 25.0–29.9 kg/m^2^ (OW), 16.6% had BMI 30.0–34.9 kg/m^2^ (OBI) and 8.1% had BMI ≥ 35.0 kg/m^2^ (OBII–III) in 2017 (Table 1). Greater age was associated with OW and OB; individuals with OW, OBI, and OBII–III were on average 45.0, 45.0 and 42.8 years old, respectively, compared with the individuals in the NW group (18.5–24.9 kg/m^2^), who were on average 38.4 years old. In the NW and OBII–III groups, over half of the individuals were female, whereas male sex was more common in the OW and OBI groups.

**Table 1:** Demographic and clinical characteristics of the main study group (working-age individuals, 18–64 years). The number of individuals refer to a weighted number in each group

BMI (kg/m^2^)	Overall	NW (18.5–24.9 kg/m^2^)	OW (25.0–29.9 kg/m^2^)	OBI (30.0–34.9 kg/m^2^)	OBII – III (35.0 + kg/m^2^)	*p*
*n* (%)	4,134 (100)	1,630 (39.4)	1,481 (35.8)	688 (16.6)	335 (8.1)	
Age (years), mean (SD)	42.2 (13.5)	38.4 (13.4)	45.0 (12.3)	45.0 (13.3)	42.8 (14.8)	< 0.001
Sex, *n* (%)						
*Female*	2,027 (49.0)	923 (56.7)	608 (41.0)	300 (43.7)	196 (58.4)	< 0.001
*Male*	2,107 (51.0)	706 (43.3)	873 (59.0)	388 (56.3)	139 (41.6)	
BMI (kg/m^2^), mean (SD)	27.2 (5.2)	22.6 (1.6)	27.3 (1.4)	32.0 (1.3)	39.1 (4.0)	< 0.001
Follow-up, years (SD)	4.7 (0.9)	4.8 (0.7)	4.7 (1.0)	4.7 (1.0)	4.7 (0.9)	< 0.001
MetCs, mean (SD)	1.18 (1.0)	0.73 (0.8)	1.24 (1.0)	1.73 (1.1)	1.93 (1.18)	< 0.001
MetCs category, *n* (%)						
* 0*	1,278 (30.9)	742 (45.5)	395 (26.7)	93 (13.6)	47 (14.0)	< 0.001
* 1 *	1,438 (34.8)	625 (38.4)	538 (36.3)	199 (28.9)	76 (22.6)	
> *1*	1,419 (34.3)	263 (16.1)	548 (37.0)	396 (57.6)	213 (63.5)	
Comorbidities, *n* (%)						
*Psychiatric disease*	952 (23.0)	349 (21.4)	311 (21.0)	210 (30.6)	82 (24.5)	0.004
*Musculoskeletal disorder*	1,981 (47.9)	684 (42.0)	726 (49.0)	380 (55.3)	191 (57.0)	< 0.001
*Metabolic disorder*	466 (11.3)	76 (4.7)	172 (11.6)	133 (19.3)	84 (25.1)	< 0.001
*Any CVD*	980 (23.7)	237 (14.6)	384 (25.9)	234 (34.0)	125 (37.3)	< 0.001
*Asthma*	275 (6.6)	98 (6.0)	84 (5.7)	47 (6.8)	46 (13.6)	0.009
*Cancer*	179 (4.3)	55 (3.4)	65 (4.4)	45 (6.5)	14 (4.1)	0.01
*Sleep apnea*	262 (6.3)	35 (2.1)	92 (6.2)	79 (11.5)	56 (16.8)	< 0.001
*T2DM*	191 (4.6)	8 (0.5)	55 (3.7)	71 (10.3)	57 (17.0)	< 0.001

*Abbreviations*: *BMI* Body mass index, *CVD* Cardiovascular disease, *MetCs* Metabolic comorbidities, *NW* Normal weight, *OBI* Class I obesity, *OBII – III* Class II – III obesity, *OW* Overweight, *SD* Standard deviation, *T2DM* Type 2 diabetes mellitus

The prevalence of MetCs increased with the increase in BMI: the proportion of individuals without MetCs was 45.5% in NW, 26.7% in OW, 13.6% in OBI, and 14.0% in OBII–III groups (Table 1). All studied disease groups were more common in individuals with OB than individuals with NW: the highest differences were observed for musculoskeletal disorders, metabolic and cardiovascular disorders, sleep apnea, and type 2 diabetes.

### Sick leaves, disability pensions and rehabilitation periods

The proportion of individuals with long (> 10 days) sick leaves, disability pensions and rehabilitation periods increased with higher BMI (Table 2). In the NW group, 26.3% of the individuals had long sick leaves, whereas the corresponding proportions were 31.3% in the OW, 33.8% in the OBI and 33.4% in the OBII-III groups (*p* = 0.024). The mean annual length of a sick leave increased from 21.9 days (SD, 29.3) in the NW group to 32.3 days (36.3) in the OBI and 35.4 days (39.8) in the OBII–III groups.

**Table 2:** Sick leaves, disability pensions, rehabilitation periods, and premature mortality in the working-age population. The number of individuals refer to a weighted number of in each group

BMI (kg/m^2^)	Overall	NW (18.5–24.9 kg/m^2^)	OW (25.0–29.9 kg/m^2^)	OBI (30.0–34.9 kg/m^2^)	OBII – III (35.0 + kg/m^2^)	*p*
*N*	4,134	1,630	1,481	688	335	
Indirect costs, *n* (%)	1,397 (33.8)	470 (28.9)	518 (35.0)	272 (39.5)	137 (40.9)	< 0.001
Long sick leaves (> 10 days), *n* (%)	1,236 (29.9)	428 (26.3)	463 (31.3)	233 (33.8)	112 (33.4)	0.024
* Total cohort, mean number of days (SD)*	8.4 (23.0)	5.8 (17.8)	9.3 (24.7)	10.9 (26.1)	11.9 (28.4)	< 0.001
*Individuals with sick leaves, mean number of days (SD)*	28.1 (34.8)	21.9 (29.3)	29.6 (36.6)	32.3 (36.3)	35.4 (39.8)	< 0.001
Disability pensions, *n* (%)	146 (3.5)	31 (1.9)	51 (3.4)	42 (6.1)	23 (6.8)	< 0.001
*Total cohort, mean number of (days) (SD)*	11.3 (64.6)	6.0 (46.2)	10.4 (60.5)	20.5 (89.7)	22.1 (89.8)	< 0.001
*Individuals with disability pensions, mean number of days (SD)*	319.1 (140.3)	315.1 (120.8)	303.9 (132.8)	337.9 (158.6)	323.8 (141.8)	0.797
Rehabilitation periods, *n* (%)	164 (4.0)	44 (2.7)	52 (3.5)	50 (7.3)	18 (5.5)	< 0.001
*Total cohort, mean number of days (SD)*	0.7 (7.9)	0.3 (5.9)	0.5 (6.2)	1.4 (10.6)	1.6 (13.7)	0.420
*Individuals with rehabilitation periods, mean number of days (SD)*	17.1 (35.7)	12.7 (34.0)	14.6 (30.2)	19.7 (34.4)	29.3 (51.1)	0.723
Premature mortality						
*Total cohort, mean number of days of life lost to due to premature mortality (SD)*	0.9 (13.3)	0.7 (11.8)	1.1 (15.1)	0.9 (11.7)	1.4 (14.9)	0.793
*Individuals with premature death, mean number of days of life lost to due to premature mortality (SD)*	142.4 (82.1)	153.7 (85.4)	160.6 (83.2)	119.5 (66.6)	107.3 (72.3)	0.547

*Abbreviations*: *BMI* Body-mass index, *NW* Normal weight, *OBI* Class I obesity, *OBII – III* Class II – III obesity, *OW* Overweight, *SD* Standard deviation

Disability pensions were observed in 1.9% of the individuals in the NW group compared with 6.1% in the OBI and 6.8% in the OBII groups, but there was no statistical difference in the mean length of a disability pension between BMI groups. The proportion of individuals with rehabilitation periods increased from 2.7% in the NW group to 7.3% and 5.5% in the OBI and OBII–III groups, respectively. There was a tendency for longer rehabilitation periods in the higher BMI groups, although the difference was not statistically significant.

### Annual total indirect costs and stratified by the cost component

**Fig. 1:**
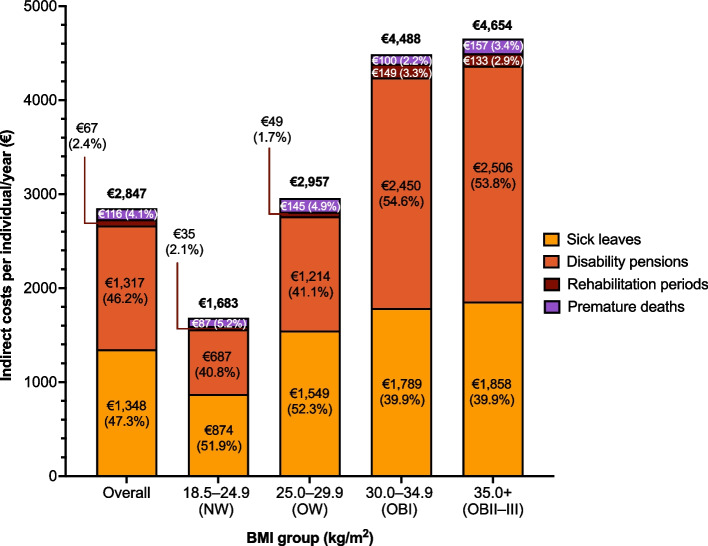
Mean annual indirect costs per person in different BMI groups in the working-age population over the whole follow-up period, stratified by cost component. BMI, body-mass index

The mean annual total indirect costs were €1,683 (SD, €6,395) per person in the NW, €2,957 (€8,797) in the OW, €4,488 (€11,607) in the OBI, and €4,654 (€11,383) in the OBII–III groups (Fig. 1, Supplementary Table 3). Overall, sick leaves and disability pensions were the main contributors (93.5%) to the total indirect costs.

The cost of all indirect components increased with the higher BMI group. In the NW and OW groups, the main component of indirect costs was sick leaves (52%), whereas disability pensions contributed most to indirect costs in the OBI (54.6%) and OBII–III (53.8%) groups. Costs of rehabilitation periods and premature mortality represented 2.4% and 4.1%, respectively, of the total indirect costs across BMI groups. Mental (ICD-10, F-codes) and musculoskeletal disorders (M-codes) were the most common causes for sick leaves, disability pensions, and rehabilitation periods in all BMI groups, and no significant differences were observed in the distribution of causes between the BMI groups.

### Annual indirect costs by sex

For men, 23% in the NW, 31% in the OW, 37% in the OBI, and 28% in the OBII–III groups had indirect costs, whereas for women, the corresponding proportions were 33% (NW), 41% (OW), 43% (OBI), and 50% (OBII–III). Considering only individuals with indirect costs, the mean total indirect costs were higher for men compared with women in all BMI groups (Supplementary Fig. 2). Excluding sick leaves, the mean costs of all other components were higher for men compared with women. For men with indirect costs, disability pension was the main contributor to total indirect costs in all BMI groups, whereas in women sick leave was the main contributor in all BMI groups except OBII–III.

### Annual total costs

**Fig. 2:**
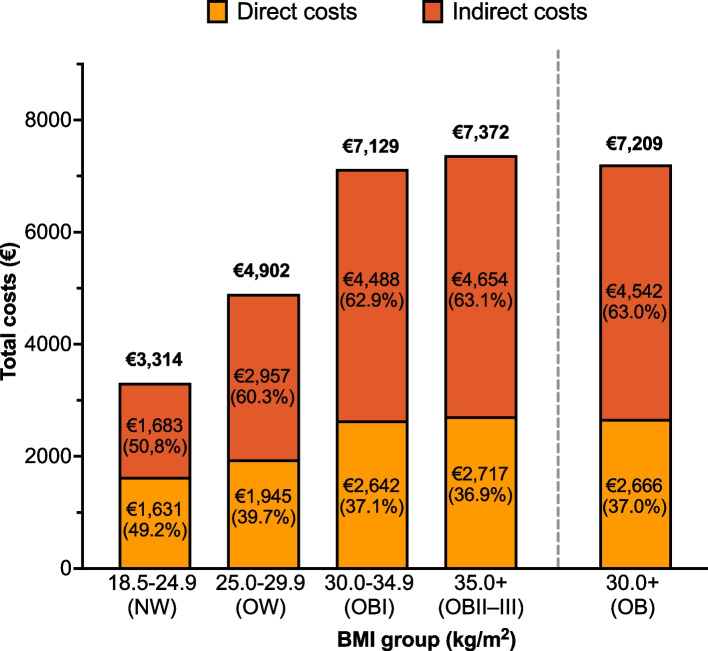
Mean annual total costs per person in different BMI groups in the working-age population over the whole follow-up period, stratified by the cost type (direct and indirect). BMI, body-mass index

In the working-age population, the mean annual total costs were €3,314 (SD, €8,358) per person in NW, €4,902 (€10,747) in OW, €7,129 (€14,313) in OBI, and €7,372 (€14,423) in OBII–III groups (Fig. 2). In the NW group, direct (49.2%) and indirect (50.8%) costs contributed almost equally to total costs, whereas in all other groups, the proportion of indirect costs was higher than of direct costs (OW, 60.3%; OBI, 62.9%; and OBII–III, 63.1%). A sensitivity analysis excluding the last year of follow-up (i.e., the year overlapping with the COVID-19 pandemic) did not show marked differences in total, indirect, and direct costs compared with the original analyses (Supplementary Table 4).

### Association between the BMI group and indirect costs

**Fig. 3:**
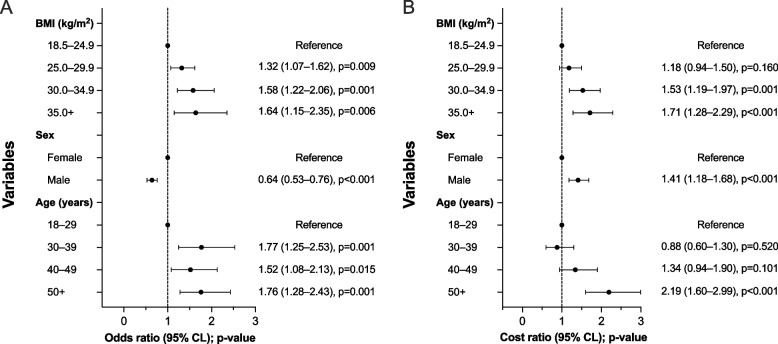
The association between the BMI groups, sex, and age group with indirect costs in the working-age population. **A** The odds ratio (95% confidence intervals) of having indirect cost estimated using the logistic regression model, and (**B**) the cost ratio (95% confidence intervals) of positive indirect costs estimated using overdispersed Poisson regression. BMI, body-mass index; CI, confidence interval

The association between the BMI group and indirect costs was assessed using two multivariate models adjusted for age and sex. In the first model, with non-zero costs as an outcome (Fig. 3A), OBI was associated with 58% (odds ratio [OR], 1.58; 95% CI, 1.22–2.06; *p* = 0.001) and OBII–III with 64% (OR, 1.64; 95% CI, 1.15–2.35; *p* = 0.006) higher risk of having indirect costs compared with individuals with NW. Overweight was associated with 32% higher risk (OR, 1.32; 95% CI, 1.07–1.62; *p* = 0.009) of having indirect costs, whereas male sex was associated with a lower risk of having indirect costs (OR, 0.64; 95% CI, 0.53–0.76; *p* < 0.001). In the second model with positive costs as an outcome (Fig. 3B), OBI was associated with 53% (cost ratio [CR], 1.53; 95% CI, 1.19–1.97; *p* = 0.001) and OBII with 71% (CR, 1.71; 95% CI, 1.28–2.29; *p* < 0.001) higher costs compared with individuals with NW. In that model, male sex was associated with higher indirect costs (CR, 1.41; 95% CI, 1.18–1.68; *p* < 0.001).

### Association between the BMI group and total costs

**Fig. 4:**
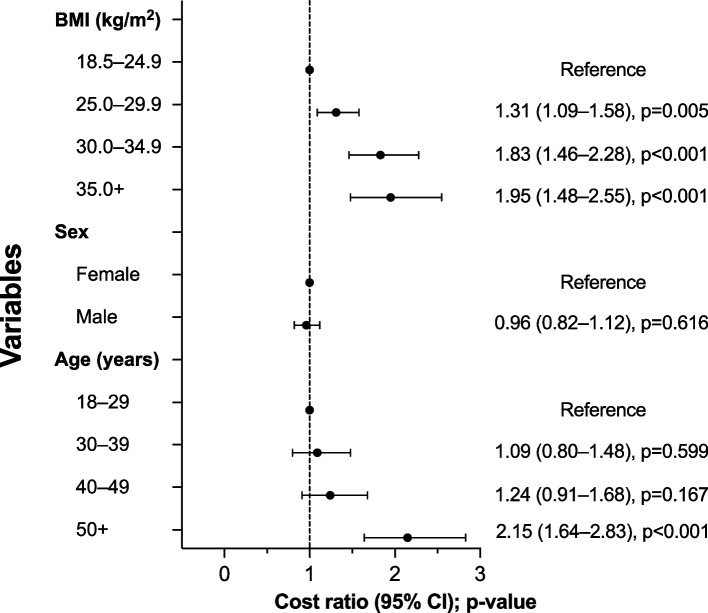
The association (cost ratios and 95% confidence intervals) between BMI groups, sex, and age group with total (direct + indirect) costs in the working-age population estimated using an overdispersed Poisson regression model. BMI, body-mass index; CI, confidence interval

Compared with individuals with NW, OW was associated with 31% (CR, 1.31; 95% CI, 1.09–1.58; *p* = 0.005), OBI with 83% (CR, 1.83; 95% CI, 1.46–2.28; *p* < 0.001), and OBII–III with 95% (CR, 1.95; 95% CI, 1.48–2.55; *p* < 0.001) higher age- and sex-adjusted total costs (Fig. 4). Similarly, OW was associated with 19% (CR, 1.19; 95% CI, 1.00–1.41; *p* = 0.050), OBI with 56% (CR, 1.56; 95% CI, 1.25–1.93; *p* < 0.001), and OBII–III with 56% (CR, 1.56; 95% CI, 1.19–2.05; *p* = 0.001) higher direct costs when compared with individuals with NW (Supplementary Fig. 3). When adjusted for age and sex, the predicted total annual cost difference was €1,124 per person with OW, €3,002 per person with OBI, and €3,443 per person with OBII–III compared with a person with NW (Supplementary Fig. 4). Overall, OB was associated with €3,146 higher costs than NW.

### Total costs by the BMI group at the national level

**Fig. 5:**
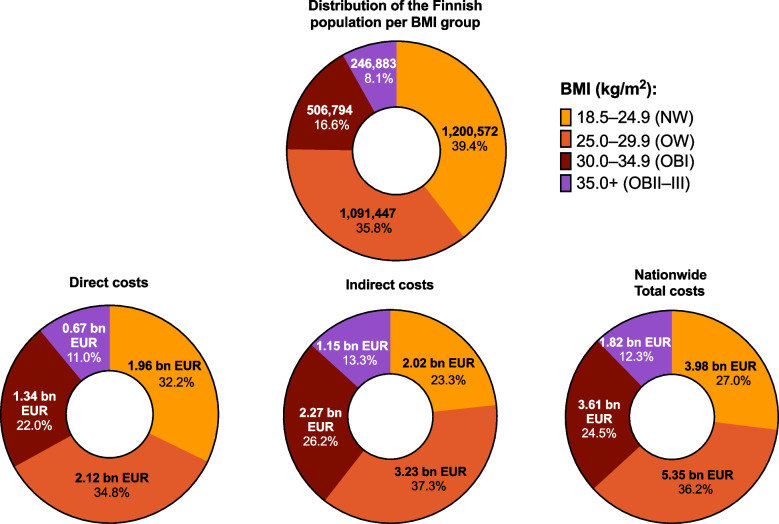
Distribution of the Finnish working-age population and costs at the national level stratified by BMI group over the whole follow-up period. BMI, body-mass index

At the national level, working-age individuals with OBI and OBII–III comprised 24.7% of the total population and were responsible for 33.0% (2.01bn), 39.5% (3.42bn), and 36.8% (5.43bn) of the direct, indirect, and total costs, respectively (Fig. 5). Individuals with OW comprised 35.8% of the population and 36.2% (5.35bn) of the total costs.

Based on the estimated number of working-age individuals in the Finnish population (*n* = 3,045,696), the extrapolated additional costs at the national-level were €1.23bn for OW (*n* = 1,091,447), €1.52bn for OBI (*n* = 506,794), and €0.85bn for OBII–III (*n* = 246,883) (Supplementary Fig. 5).

### Direct, indirect, and total costs in the total adult Finnish population

A supplementary analysis was performed to estimate direct, indirect, and total costs of obesity in the total adult (≥ 18 years) population in Finland (*n* = 4,074,592). The mean annual total costs were €3,535 (SD, €7,935) per person in NW, €4,982 (€9,736) in OW, €6,362 (€11,597) in OBI, and €6,868 (€11,858) in OBII–III groups (Supplementary Fig. 6). When adjusted for age and sex, the predicted total annual total cost difference was €1,036 per person for OW, €2,221 per person for OBI, and €2,896 per person for OBII–III when compared with a person with NW (Supplementary Fig. 4). At the national level, the additional costs were €1.62bn for OW (*n* = 1,564,800), €1.62bn for OBI (*n* = 728,884), and €0.92bn for OBII–III (*n* = 317,615) (Supplementary Fig. 5).

## Discussion

This nationally representative, population-based study utilized individual-level data from the biobank and health and social care registers to assess indirect, direct, and total costs of overweight and obesity in Finland. The results indicated that in working-age individuals, indirect costs were 1.5-fold and 1.7-fold higher than direct costs in OW and OB, respectively; in contrast to individuals with NW, who had almost equal direct and indirect costs. Compared with individuals with NW, age- and sex-adjusted annual total (direct + indirect) costs were 31% (€1,124), 83% (€3,002), and 95% (€3,443) higher for individuals with OW, OBI, and OBII–III, respectively.

At the national level, total additional costs associated with adult overweight and obesity were €1.6bn and €2.5bn, respectively. This corresponds to 1.5% of the gross domestic product (GDP), exceeding the estimated costs of alcohol (€1.4bn annually) and tobacco (€1.3bn in 2020) consumption. The proportion of GDP is lower than the estimated average economic impact (2.2% of global GDP) of OW and OB across 161 countries in the world [29]. However, the numbers are not directly comparable due to different methodologies, data sources, and cost components included [30, 31].

Sick leaves and disability pensions were the main components (94% across the BMI groups) of indirect costs in all BMI groups, but their contribution was different in individuals with NW and OW compared with individuals with OB. The rise in indirect costs in OB was mainly driven by the significantly higher proportion of individuals with OB having disability pensions (OBI, 6.1%; OBII–III, 6.8%) compared with individuals with NW (1.9%), whereas the mean length of a disability pension was similar in all BMI groups. Correspondingly, the mean annual costs of disability pensions were approximately 3.6-fold and 3.7-fold higher for individuals with OBI (2,450€) and OBII–III (2,506€), compared with individuals with NW (687€).

The importance of this finding is highlighted by the fact that return to work after temporary disability pension has been reported to be relatively uncommon, meaning that cumulative costs over time will be considerable [32]. Thus, preventing permanent disability should be of high priority in health policies and actions to diminish the negative consequences of excess weight. Although the higher mean age in the OBI and OB II–III groups compared with individuals with NW can partly explain the remarkable difference in the prevalence of disability pensions between the BMI groups, the age-and sex adjusted indirect cost models indicate the economic burden associated with disability in individuals with obesity. Notably, the proportion of individuals with disability pensions was higher and costs approximately double in individuals with obesity also compared with individuals with OW, who had a similar mean age as individuals with OB, suggesting that the transition from overweight to obesity is associated with increased disability in the working-age population.

Although the costs of sick leaves increased relatively less with the higher BMI group than the costs of disability pensions, sick leaves were also more common, and the mean length of a sick leave longer in individuals with OB compared with NW. This is line with our previous findings using the same cohort and showing that individuals with OBI and OBII had more self-reported sick leave days than individuals with NW, and with many previous reports [6, 11–13, 18]. However, it should be noted that only long (> 10 days) sick leaves were included in the analyses, because short sick leaves are paid by the employer and not available from the registers used in this study. Short sick leaves have been estimated to account for approximately two thirds of all sick leaves and 24% of their total costs in Finland [33]. In addition, we did not assess the costs of presenteeism, which have been estimated to equal to or even surpass the costs of absenteeism [34, 35]. Indeed, our previous study indicated that individuals with OB rated their physical and psychological working ability lower compared with individuals with NW [6]. Thus, the numbers provided here likely underestimate the actual costs of reduced work productivity and hence the total indirect costs of obesity.

Interestingly, the association between sex and indirect costs seemed to be two-dimensional. Having indirect costs was more common in women, but the mean indirect costs were higher for men than women. That was mainly due to fact that disability pensions, which are rare but costly, dominated indirect costs in men, whereas sick leaves were the main cost contributor in women. When total costs were assessed, no significant association was observed between the sexes.

Our results showed that, not only OB, but also OW was associated with significantly higher total costs compared with NW in working-age individuals, in contrast to our previous findings which only assessed direct costs in the adult population [9, 25]. In addition, the effect size in direct costs between individuals with OB and the NW was bigger in the working-age population than in our previous study. This suggests that the direct healthcare burden of OW and OB is higher in the working-age population compared with elderly. Indeed, obesity in older adults has been proposed to be more complex than in young and middle-aged adults, and an ideal BMI range may be different [36, 37].

One of the possible explanations for a different association between OB and direct costs in the working-age and total adult population could be that in the aging population, individuals with highly prevalent multimorbidity, incurring the highest costs, have passed away. Overall, multimorbidity has been shown to cause a heavy burden on Finnish healthcare [38]. Previously, we showed that the attributable direct costs of obesity were mainly driven by the increased prevalence of metabolic comorbidities in individuals with OB [9, 25]. Only approximately 10% of individuals with OB were metabolically healthy, and individuals with OB had higher HCRU and medication use in all disease groups. Here, the main contributors (mental and musculoskeletal diseases) to indirect costs were similar in all BMI groups, suggesting that the increase in indirect costs with OB is expected to be largely due to significantly increased overall morbidity.

The main strengths of this study are the nationally representative sample of Finnish adults, and comprehensive data collected from several national health and social care registers, which allowed a detailed and reliable estimation of both indirect and direct costs at the population level. The BMIs for the study individuals were measured in clinical examination, in contrast to self-reporting which has the tendency of underreporting weight and overreporting height [39]. Limitations include the lack of information on short sick leaves and presenteeism in indirect costs, and the lack of private and occupational healthcare in national registers before the year 2019. In addition, longitudinal data on unemployment or shortfall in labour force participation were not available in the registers used. Overall, estimating exact indirect costs and the true value of lost work productivity is challenging, as the percentage of labor force participation can vary between age groups for different reasons including, e.g., studies or parental leaves.

Variation in costs was high even within the BMI group, and the relatively small number of individuals especially in the OBII–III group caused wide confidence intervals. It should also be noted that individuals with a weak physical condition are more likely to opt-out of health examinations and thus the study population, which may cause bias especially in high BMI classes. In addition, modelling results can somewhat depend on the approach used. For the result of predicted total annual cost difference per person, we tested different types of models (Gamma, inverse Gaussian) in sensitivity analyses. The original Poisson result for excess cost was €3,443 for OBII–III compared with a person with NW. With different models, the corresponding result was quite stable (€3,370–€3,448). There was more variation in the predicted absolute value in those with OBII-III (€6,868–€7,183). This gives insight that the excess costs are quite stable, whereas the predicted absolute values are more prone to model selection. Noteworthy, complex, multi-directional relationships have been shown between obesity and many of the assessed factors. As the relationship between many chronic diseases, such as musculoskeletal and mental disease, and excess weight is bi-directional, reverse causality between obesity and e.g. disability pensions cannot be ruled out [40–42]. Obesity is also known to be associated with educational level and socioeconomic status, which in turn have a complex interplay with sickness absence [43–45]. Due to these reasons, the aim of this paper was not to show direct causality or factors mediating the effect of increasing BMI on costs, but instead characterize the overall burden of excess weight in real life.

It should also be pointed out that seasonal phenomena, like the COVID-19 pandemic can potentially affect individuals with normal-weight vs. overweight or obesity differently, which in turn is reflected on the costs. However, in our sensitivity analyses we were not able to see major differences in mean annual costs for any of the cost components when excluding the last year of the study period, which overlapped with the COVID-19 pandemic. To address the association between obesity and costs related to COVID-19 pandemic, more detailed studies analyzing the years over the whole period of COVID-19 pandemic separately would be needed.

Together, these findings show that indirect costs constitute a major part of the costs of obesity in the working-age population and strengthens the importance of thinking beyond the cost of direct medical care when evaluating the economic impact of obesity. Indirect costs resulting from disability and sickness are also associated with human suffering, and actions to reduce the cost burden go along with improved health status and quality of life. The burden associated with obesity is not restricted to individuals with severe obesity (OBII–III), but a significant increase in costs was observed also with OBI, indicating that negative health impacts accumulate when the BMI rises ≥ 30 kg/m^2^. Health programs and policies that focus on systemic solutions that tackle the root causes of overweight and obesity, as well as sufficient support and treatment for people living with obesity, are required to reduce the negative economic impacts.

## Supplementary Information

Supplementary Material 1.

## Data Availability

The datasets generated and/or analyzed during the current study are not publicly available according to the Finnish legislation, as access to individual-level data is restricted only to individuals named in the study permit. The study protocol is available upon request from the corresponding author.

## Abbreviations

ATP III: National Cholesterol Education Program’s Adult Treatment Panel III report
BMI: Body mass index
CI: Confidence interval
CVD: Cardiovascular disease
GDP: Gross domestic product
HCA: Human Capital Approach
HCRU: Healthcare resource utilization
ICD-10: International Classification of Diseases – 10th revision
Kela: The social insurance institution of Finland
MetCs: Metabolic comorbidities
NW: Normal weight
OB: Obesity
OBI: Class I obesity
OBII–III: Class II–III obesity
OW: Overweight
QoL: Quality of life
RR: Rate ratio
SD: Standard deviation
THL: The Finnish institute for health and welfare
T2DM: Type 2 diabetes mellitus
