# Supplementary material for: Indirect costs constitute a major part of the total economic burden of obesity: a Finnish population-based cohort study
Source: BMC Public Health. 2025 May 10;25:1739. doi: 10.1186/s12889-025-22978-9 (PMC12065301; doi:10.1186/s12889-025-22978-9)
Supplement: Supplementary file 1 — Supplementary Material 1. [file 12889_2025_22978_MOESM1_ESM.docx]

**Supplementary information**

**Indirect costs constitute a major part of the total economic burden of obesity: A Finnish population-based cohort study**

Aino Vesikansa^1^, Juha Mehtälä^1*^, Susanna Aspholm^2^, Kirsi Kallio-Grönroos^2^, Katja Mutanen^2^, Annamari Lundqvist^3^, Tiina Laatikainen^3,4,5^, Tero Saukkonen^2^, Kirsi H. Pietiläinen^6^

^1^MedEngine Oy, Helsinki, Finland

^2^Novo Nordisk Farma Oy, Espoo, Finland

^3^Finnish Institute for Health and Welfare, Helsinki, Finland

^4^Institute of Public Health and Clinical Nutrition, University of Eastern Finland, Kuopio, Finland

^5^Joint Municipal Authority for North Karelia Social and Health Services (Siun Sote), Joensuu, Finland

^6^Obesity Research Unit, University of Helsinki, Helsinki, Finland

***Corresponding Author:**

Juha Mehtälä

Eteläranta 14

00100 Helsinki, Finland

Email: [juha.mehtala@medengine.fi](mailto:juha.mehtala@medengine.fi)

Tel. +358 40 773 5059

**SUPPLEMENTARY TABLES**

**Supplementary Table 1.** Description of registry data sources. Registry data were linked with the FinHealth 2017 data using personal identification numbers.

| **Register** | **Description** |
| --- | --- |
| The Care Register for Healthcare (secondary healthcare)  and  The Register for Primary Healthcare Visits (primary healthcare) | **Register holder:** Finnish Institute for Health and Welfare (THL)  **Variables collected in this study:**   - HCRU based on the service provider and field of service (outpatient contacts, inpatient periods) - Diagnoses and comorbidities (ICD-10 and ICPC-2) - Operations based on NCSP coding and associated costs |
| The Prescription Register | **Register holder:** The Social Insurance Institution of Finland (Kela)  **Variables collected in this study:**   - Medication use (purchases of all reimbursed medications) - Data on the drug (ATC code) - Medication costs |
| Social Benefits Registers by The Social Insurance Institution of Finland:   - *Kelan maksetut eläke-etuudet (MAKY) Kela's paid pension benefits (MAKY)* - *Kelan eläke-etuuksien saajat*   *(ASN) / National Pension Fund / Recipients of Kela's pension benefits*  *(ASN)*   - *Kuntoutusrahan saajat ja*   *maksetut etuudet (UMA) / Recipients of rehabilitation allowance and*  *paid benefits (UMA)*   - *Sairauspäivärahojen maksut*   *(SMA) / Sickness allowance payments*  *(SMA)*   - *Sairauspäivärahojen maksetut*   *Kaudet / Sickness benefits paid*  *Seasons* | **Register holder:** The Social Insurance Institution of Finland (Kela)  **Variables collected in this study:**   - Sick leaves: dates, causes (ICD-10) - Disability pensions: dates, causes (ICD-10) - Rehabilitation periods: dates, causes (ICD-10) |
| Cause of Death Register | **Register holder:** Statistics Finland  **Variables collected in this study:**   - Dates and causes (ICD-10) of death |

**Abbreviations**: ATC, Anatomical Therapeutic Chemical; HCRU, healthcare resource utilization; ICD-10, International Classification of Diseases – 10^th^ Revision; ICPC-2, International Classification of Primary Care 2; Kela, The Social Insurance Institution of Finland; NCSP, NOMESCO Classification of Surgical Procedures; THL, Finnish Institute for Health and Welfare.

**Supplementary Table 2.** Definitions of comorbidity groups related to healthcare resource utilization by their International Classification of Disease, 10^th^ revision codes (ICD-10). Based on The Care Register for Healthcare and The Register for Primary Healthcare Visits by The Finnish Institute for Health and Welfare.

| **Primary cause of visit** | **ICD-10** |
| --- | --- |
| Psychiatric disorder | F00–F99 |
| Musculoskeletal disorders | M05 – M99 |
| Metabolic disorders | E11, R73, E78, K73.8, K74, K76.0, K76.1 |
| Any cardiovascular diseases (CVD) | I10 – I99 |
| Asthma | J45, J46 |
| Cancer | C00 – C99 |
| Sleep apnea | G47.3 |
| Type 2 Diabetes mellitus (T2DM) | E11 |

**Supplementary Table 3.** Annual direct, indirect, and total costs per cost component in different BMI groups in the Finnish working-age population.

| **BMI (kg/m^2^)** | **Overall** | **NW**  **(18.5-24.9 kg/m^2^)** | **OW**  **(25.0-29.9 kg/m^2^)** | **OBI**  **(30.0-34.9 kg/m^2^)** | **OBII – III**  **(35.0+ kg/m^2^)** | ***p*** |
| --- | --- | --- | --- | --- | --- | --- |
| **Direct costs (€), mean (SD)** | **2,000 (4,162)** | **1,631 (3,635)** | **1,945 (4,101)** | **2,642 (4,861)** | **2,717 (4,974)** | **<0.001** |
| *Primary healthcare* | 447 (1,289) | 363 (477) | 435 (1,724) | 550 (1,114) | 702 (1,896) | <0.001 |
| *Secondary healthcare* | 1,102 (2,986) | 955 (2,949) | 1,046 (2,718) | 1,452 (3,353) | 1,346 (3,410) | 0.020 |
| *Prescribed medications* | 451 (1,533) | 313 (1,099) | 464 (1,786) | 640 (1,776) | 669 (1,536) | <0.001 |
| **Indirect costs (€), mean (SD)** | **2,847 (8,829)** | **1,683 (6,395)** | **2,957 (8,797)** | **4,488 (11,607)** | **4,655 (11,383)** | **<0.001** |
| *Sick leaves* | 1,348 (3,753) | 874 (2,805) | 1,549 (4,124) | 1,789 (4,316) | 1,858 (4,502) | <0.001 |
| *Disability pensions* | 1,317 (7,568) | 687 (5,304) | 1,214 (7,153) | 2,450 (10,661) | 2,506 (10,243) | <0.001 |
| *Rehabilitation periods* | 67 (752) | 35 (618) | 49 (602) | 149 (1,070) | 134 (1,069) | 0.420 |
| *Deaths* | 116 (1,667) | 87 (1,507) | 145 (1,970) | 100 (1,321) | 157 (1,588) | 0.812 |
| **Total (direct + indirect) (€),**  **mean (SD)** | **4,847 (11,048)** | **3,314 (8,358)** | **4,902 (10,747)** | **7,129 (14,313)** | **7,372 (14,423)** | **<0.001** |

**Abbreviations**: BMI, body-mass index; NW, normal weight; OBI, class I obesity; OBII – III, class II – III obesity; OW, overweight; SD, standard deviation.

**Supplementary Table 4.** Mean annual direct, indirect, and total costs between 1^st^ of April 2016 until 31^st^ March 2021 (original analysis) and between 1^st^ of April 2016 until 31^st^ March 2020 (excluding the first year of the COVID-19 pandemic) in the Finnish working-age population.

| **BMI (kg/m^2^)** | **Total costs (€)** | **Indirect costs (€)** | **Direct costs (€)** |
| --- | --- | --- | --- |
| Original analysis (1st of April 2016 until 31st March 2021) | | | |
| **18.5–24.9** | 3,314 | 1,683 | 1,631 |
| **25.0–29.9** | 4,902 | 2,957 | 1,945 |
| **30.0–34.9** | 7,129 | 4,488 | 2,642 |
| **35.0+** | 7,372 | 4,654 | 2,717 |
| Last year removed (1st of April 2016 until 31st March 2020) | | | |
| **18.5–24.9** | 3,268 | 1,652 | 1,615 |
| **25.0–29.9** | 4,657 | 2,791 | 1,865 |
| **30.0–34.9** | 6,901 | 4,363 | 2,538 |
| **35.0+** | 7,053 | 4,329 | 2,723 |

**SUPPLEMENTARY FIGURES**

**Supplementary Figure 1.** Absolute and weighted number of observations in the Finnish working-age and total adult populations derived from the FinHealth 2017 cohort.

**Supplementary Figure 2**. Mean annual indirect costs per person over the whole follow-up period in different BMI groups for the Finnish working-age population: A) male individuals, and B) female individuals who had non-zero indirect cost, stratified by the cost component. BMI, body-mass index.

**Supplementary Figure 3.** The association (cost ratios and 95% confidence intervals) between BMI groups, sex, and age groups with direct costs in the Finnish working-age population estimated using an overdispersed Poisson regression model. BMI, body-mass index; CI, confidence interval.

**Supplementary Figure 4**. Age- and sex-adjusted additional direct, indirect, and total costs per person per year in different BMI groups as compared with individuals with normal weight for direct, indirect, and total costs in the Finnish working-age and total adult populations.

**Supplementary Figure 5.** Extrapolated additional adjusted direct, indirect, and total costs at the national level in Finland in different BMI groups compared with individuals with normal weight.

**Supplementary Figure 6.** Mean annual total costs per person in different BMI groups in the Finnish total adult population over the whole follow-up period, stratified by the cost type (direct and indirect). BMI, body-mass index.
